# Utilization of artificial intelligence in prostate cancer detection: a comprehensive review of innovations in screening and diagnosis

**DOI:** 10.3389/fimmu.2025.1670671

**Published:** 2025-11-27

**Authors:** Emad Rajih, Abdulaziz Bakhsh, Walaa M. Borhan, Saeed Awad M. Alqahtani

**Affiliations:** 1 General and specialized surgery department, College of Medicine, Taibah University, Medina, Saudi Arabia; 2 Basic Medical Sciences Department, College of Medicine, Taibah University, Medina, Saudi Arabia

**Keywords:** artificial intelligence, prostate cancer, medical imaging, deep learning, personalized medicine

## Abstract

Prostate cancer management has long been challenged by the limitations of traditional screening tools like PSA testing, which contribute to significant rates of overdiagnosis and overtreatment. While advanced imaging such as multiparametric MRI (mpMRI) has improved the diagnostic pathway, the integration of Artificial Intelligence (AI) is now catalyzing a paradigm shift across the entire continuum of care. This comprehensive review details the transformative role of AI in prostate cancer. In diagnostics, deep learning algorithms enhance the interpretation of mpMRI by improving lesion detection, segmentation, and risk stratification, thereby reducing unnecessary biopsies. In digital pathology, AI provides automated and consistent Gleason grading, minimizing inter-observer variability and refining prognostication. In the therapeutic domain, AI is crucial for personalizing treatment by streamlining radiotherapy planning through automated contouring, predicting patient outcomes and toxicity, and enabling the development of adaptive therapy strategies for advanced disease. Multimodal AI models that synthesize imaging, biomarker, and clinical data are creating robust predictive tools for superior clinical decision support. Despite formidable challenges related to prospective validation, data equity, and regulatory approval, AI is paving the way for a new standard of care characterized by greater precision, efficiency, and personalization.

## 1 Introduction

Global cancer data from GLOBOCAN indicates that in 2022, prostate cancer accounted for an estimated 1.47 million new cases and 400,000 deaths globally, persisting as the most diagnosed cancer among men in many nations and a leading cause of cancer mortality (1). The global burden is rising with ageing populations and widening geographic disparities, underscoring the need for more precise, efficient diagnostic pathways. Prostate cancer presents a complex clinical spectrum from slow-growing, indolent tumors that may never require intervention to aggressive, life-threatening diseases. For decades, the clinical approach to its detection and management has been built upon a foundation of established, albeit imperfect, tools. The evolution of this field is a story of continuous refinement, driven by a quest for greater precision, reduced patient harm, and more personalized care. This journey has progressed from broad population screening to advanced molecular and imaging techniques and now stands at the cusp of a new era defined by the integration of Artificial Intelligence (AI).

The cornerstone of prostate cancer screening for over three decades has been the Prostate-Specific Antigen (PSA) test. The principal advantages of PSA testing are its non-invasive nature, low cost, and widespread availability, making it an accessible first-line screening tool. However, its utility is severely hampered by its low specificity for clinically significant cancer. Elevated PSA levels are not exclusive to malignancy; they are frequently caused by benign conditions such as benign prostatic hyperplasia, a common non-cancerous enlargement of the prostate, or prostatitis, an inflammation of the gland (2, 3). This lack of specificity is the primary driver of the test’s main controversy: the substantial risk of overdiagnosis—the detection of indolent cancers that would never have caused symptoms or death—and consequent overtreatment, subjecting men to invasive procedures with life-altering side effects like incontinence and erectile dysfunction (2, 4).

Complementing the PSA test is the Digital Rectal Examination (DRE) which is inexpensive and can occasionally detect aggressive cancers in men with normal PSA levels. However, its diagnostic value is highly subjective, depending heavily on the examiner’s experience, the patient’s anatomy, and the tumor’s size and location. Cancers located in the anterior part of the prostate are typically inaccessible to palpation. Consequently, the DRE suffers from low sensitivity and specificity and is no longer recommended by some guidelines as a standalone screening tool, but rather as an adjunct to PSA testing (5, 6).

When screening tests suggest a risk of cancer, the definitive gold standard for diagnosis has historically been the systematic transrectal ultrasound (TRUS)-guided prostate biopsy. While this method provides a definitive histological diagnosis, it is an invasive procedure with inherent risks, including bleeding, infection, pain, and urinary difficulties (7). More importantly, this “blind” systematic approach can miss significant tumors or underestimate the true grade of the disease, as the small core samples may not be representative of the most aggressive part of the tumor.

To address these limitations, emerging biomarkers and liquid-based assays—such as the Prostate Health Index (PHI), 4Kscore Test, Prostate Cancer Gene 3 (PCA3) test, and liquid biopsies have been introduced to complement the traditional biopsy pathway (8–12), offering varying strengths and limitations in improving risk stratification and reducing unnecessary procedures (**Table 1**).

**Table 1:** Overview of selected biomarkers for prostate cancer risk stratification.

Biomarker test	Sample type	Key components measured	Clinical utility
PHI	Blood (Serum)	Total PSA, free PSA (fPSA), and proPSA (-2 isoform)	Combines the three markers into a single score to better predict overall and high-grade prostate cancer, reducing unnecessary biopsies (8, 9).
4Kscore Test	Blood (Serum)	Total PSA, fPSA, intact PSA, and human kallikrein 2	Calculates the percentage risk of having aggressive prostate cancer upon biopsy, aiding in the decision to proceed with an invasive procedure (10).
PCA3	Urine (post-DRE)	Measures the overexpression of the PCA3 gene, which is highly specific to prostate cancer cells.	Helps to stratify risk in men with elevated PSA, particularly in the repeat biopsy setting, as it is not affected by prostate volume or benign prostatic hyperplasia (11).
Liquid Biopsies	Blood (Plasma)	Circulating Tumor Cells, cell-free DNA, or exosomes.	A rapidly evolving field used to detect tumor-specific genetic mutations for diagnosis, prognosis, and monitoring treatment response non-invasively (12).

Simultaneously, the field of medical imaging underwent a paradigm shift with the rise of multiparametric Magnetic Resonance Imaging (mpMRI). Unlike ultrasound, mpMRI provides exquisite anatomical detail and functional information about tissue characteristics. Its high negative predictive value is particularly valuable, as a negative mpMRI result can give patients and clinicians confidence to safely avoid an immediate biopsy (13). However, its application is constrained by high costs, long acquisition times, and limited availability in many regions. Furthermore, the interpretation of mpMRI scans requires extensive sub-specialized training and suffers from considerable inter-reader variability, even among experts (14).

Into this complex landscape, AI has emerged as a transformative force, promising to augment, streamline, and enhance nearly every aspect of the prostate cancer pathway. AI, particularly in the form of machine learning and deep learning, offers the ability to analyze vast and complex datasets—from clinical parameters and biomarkers to medical images and genomic profiles—to identify patterns and make predictions that are often beyond the capability of human cognition. In imaging, AI algorithms can assist radiologists by automatically segmenting the prostate, highlighting suspicious areas, and quantifying lesion characteristics, with the primary goal of improving diagnostic accuracy while reducing inter-operator variability (15). A recent meta-analysis confirms that AI-powered decision support systems can significantly enhance the diagnostic accuracy of mpMRI across various experience levels of radiologists (16). In a broader sense, AI can integrate these disparate data sources to build personalized risk models, predict treatment outcomes, and guide therapeutic strategies, truly ushering in the era of personalized medicine (17).

This review evaluates how AI addresses key gaps in the prostate cancer pathway, with emphasis on (i) imaging (lesion detection, segmentation, staging, radiomics), (ii) digital pathology (cancer detection and Gleason grading), and (iii) clinical decision support (risk stratification, biopsy guidance, radiotherapy planning, and adaptive strategies). We synthesize evidence for clinical utility, outline implementation challenges, and highlight priorities for prospective validation and equitable deployment.

## 2 The role of AI in prostate imaging

Medical imaging is the visual core of modern prostate cancer diagnosis, and AI is fundamentally reshaping how these images are acquired, analyzed, and interpreted. AI algorithms are being developed to not only replicate but, in some cases, exceed human performance in key diagnostic tasks, promising a future of more accurate, consistent, and efficient radiological practice.

### 2.1 Enhancing MRI for lesion detection, characterization, and staging

The interpretation of mpMRI is a complex task. Radiologists must synthesize information from multiple sequences: high-resolution T2-weighted images providing anatomical context, Diffusion-Weighted Imaging highlighting areas of restricted water movement characteristic of dense cellularity found in tumors, and the Apparent Diffusion Coefficient map which quantifies this restriction. Deep learning models, particularly Convolutional Neural Networks (CNNs), are exceptionally well-suited for this type of multi-channel image analysis (18).

CNNs operate by applying a series of filters (or kernels) to input images to learn a hierarchy of features. Early layers in the network might learn to detect simple edges and textures, while deeper layers combine these to recognize more complex structures, shapes, and patterns indicative of cancerous lesions (19). Architectures like the U-Net, which excels at biomedical image segmentation, and 3D CNNs, which can process the entire volumetric data of an MRI scan at once, are widely employed (20). These models can be trained on thousands of expertly annotated scans to automatically perform critical tasks like delineating the prostate gland boundaries and segmenting suspicious lesions (21, 22). The output can be presented to the radiologist as a “bounding box” or a colored overlay on the scan, drawing their attention to areas of concern.

The performance of these models has been striking. In some studies, AI has demonstrated diagnostic performance for clinically significant cancer that is comparable to that of sub-specialist radiologists (23). One prominent study found that a U-Net-based model achieved 78% accuracy in localizing lesions, which was significantly higher than the 55% accuracy achieved by a group of non-specialist radiologists, highlighting AI’s potential to standardize and elevate care, especially in non-expert settings (24). Other advanced architectures like the MiniSegCaps network have also shown superior performance in both segmenting lesions and automatically assigning a Prostate Imaging-Reporting and Data System (PI-RADS) score, a standardized 1-to-5 scale of suspicion used by radiologists (25). By processing the entirety of the mpMRI data, these models have demonstrated the ability to distinguish malignant from benign tissues with high accuracy, achieving area under the curve (AUC) values—a measure of diagnostic performance—of up to 0.91 in recent validations (26, 27).

### 2.2 Improving ultrasound-based detection and targeting

While MRI is the premier imaging modality, its cost and accessibility are limited. TRUS is far more ubiquitous but has traditionally been poor at visualizing tumors, as many cancers are “isoechoic,” meaning they have the same texture as surrounding healthy tissue. AI is breathing new life into this modality. Deep learning algorithms can be trained to perceive subtle textural and pattern-based differences in B-mode ultrasound images that are invisible to the human eye. For instance, systems using sophisticated CNNs have been shown to differentiate between benign and malignant prostate tissue on standard TRUS images with a sensitivity of 86.23% and a specificity of 92.11% (28).

Beyond B-mode, AI is also being applied to more advanced ultrasound techniques like elastography (which measures tissue stiffness) and contrast-enhanced ultrasound. AI-assisted TRUS has already demonstrated the ability to outperform human readers in detecting clinically significant prostate cancer (csPCa) (29). The implications are profound: a more intelligent, AI-enhanced ultrasound could serve as a powerful triage tool, improving cancer detection in community settings and potentially reducing the reliance on expensive MRI. Furthermore, AI is critical for improving MRI-US fusion biopsy, where a pre-procedural MRI showing the tumor is fused with real-time ultrasound to guide the biopsy needle. AI can automate and improve the accuracy of the image registration process, ensuring that the needle is guided to the true location of the tumor identified on the MRI, an area of active and promising research (30).

### 2.3 Radiomics and multimodal AI for integrated diagnostics

Radiomics represents a paradigm of extracting vast amounts of quantitative data from medical images, moving beyond what the human eye can see. The underlying hypothesis is that these quantitative features contain latent information about tumor biology and behavior. The process involves segmenting a region of interest (the tumor) and then applying algorithms to extract hundreds or even thousands of features. These can be categorized as: first-order (describing the distribution of pixel intensities, e.g., mean, skewness, kurtosis), second-order (describing textural patterns and spatial relationships between pixels, e.g., contrast, correlation, entropy), and higher-order features derived from applying filters like wavelets.

AI, specifically machine learning, is essential for making sense of this high-dimensional feature space. Models can be trained on these radiomic features to predict outcomes like the presence of cancer, Gleason grade, or the likelihood of recurrence. Studies have shown that models built on MRI radiomic features can accurately differentiate malignant from benign tissues and are effective in predicting cancer risk and aggressiveness (31, 32). A 2024 systematic review highlighted that radiomics-based models consistently improve the prediction of extraprostatic extension and seminal vesicle invasion compared to clinical models alone, offering crucial information for surgical planning (33).

The true pinnacle of diagnostic AI lies in multimodal models that integrate diverse data streams. A radiologist interpreting an MRI does so with knowledge of the patient’s PSA level, age, and biopsy history. Similarly, AI models that combine these clinical parameters with imaging data consistently outperform models that rely on a single data source alone. For example, an AI model might learn that a lesion with ambiguous imaging features is much more likely to be significant cancer if the patient also has a high PSA density (PSA level divided by prostate volume). This integration of complementary data sources is powerful, with combined models achieving very high diagnostic accuracy and AUC values in external validations (34, 35). This holistic approach more closely mimics expert clinical reasoning, leveraging all available information to arrive at the most accurate conclusion.

AI is fundamentally reshaping prostate imaging by enhancing both high-resolution mpMRI and more common ultrasound technologies. Deep learning models, particularly CNNs, are improving the accuracy and consistency of lesion detection, segmentation, and scoring on MRI scans, in some cases matching expert-level performance. Simultaneously, AI is boosting the capabilities of ultrasound by detecting subtle tumor patterns invisible to the human eye and improving the precision of MRI-US fusion biopsies. Beyond direct image interpretation, AI enables the field of radiomics—extracting vast, invisible quantitative data from scans—and powers multimodal models that integrate imaging features with clinical data like PSA levels to predict cancer aggressiveness, mimicking expert reasoning for a more holistic and accurate diagnosis.

## 3 AI Applications in the diagnostic and screening pathway

Building on the technical capabilities summarized in Section 2, this section focuses on how AI outputs are used at key clinical decision points to improve triage, diagnostic certainty, and early treatment planning—without revisiting model architectures or image-processing mechanics.

## 3.1 AI-driven risk stratification and biopsy triage

At the pre-biopsy decision point, non-PSA biomarkers—most notably PHI, 4Kscore, PCA3, and selected liquid-biopsy assays—provide complementary information alongside mpMRI and AI-derived risk scores, supporting shared decision-making about whether to proceed to biopsy (**Table 1**). In parallel, AI models that integrate clinical variables, PSA-derived metrics, and quantitative imaging features (radiomics and other multimodal inputs) produce calibrated risk scores that guide biopsy decisions; across multiple cohorts these approaches have demonstrated high discrimination and, in some studies, have outperformed traditional clinical calculators in identifying patients at increased risk of aggressive disease (31, 32). Performance can be further strengthened by incorporating advanced MRI-derived metrics; for example, diffusion basis spectrum imaging features combined with AI have accurately predicted csPCa, and when these AI-derived scores are used alongside standard PI-RADS assessments, they can raise diagnostic confidence and support more accurate triage decisions (23, 36). Clinically, the principal utility is the potential to reduce unnecessary biopsies while preserving detection of significant cancers, thereby decreasing procedure-related harms and anxiety (23, 36). In practice, sites often implement threshold-based pathways (e.g., proceeding to biopsy above a predefined AI+PI-RADS risk level and otherwise monitoring), with periodic calibration checks to maintain performance.

### 3.2 AI in histopathological analysis

Once biopsy is performed, digital pathology becomes critical to definitive diagnosis and grading. AI systems applied to whole-slide images can rapidly highlight microscopic cancer foci and classify tissue with high accuracy in study settings, facilitating case review and prioritization (37). Importantly, AI assistance has demonstrated performance at or approaching expert levels for Gleason grading and can reduce the well-recognized inter-observer variability that affects prognostically meaningful distinctions, such as between Grade Group 2 (3 + 4) and Grade Group 3 (4 + 3) (38, 39). A recent large-scale study confirmed that an AI-based grading system demonstrated non-inferiority to expert uropathologists, suggesting its potential to serve as a reliable “second reader” and standardize quality (40). Validation across diverse international cohorts further supports the generalizability of these approaches and their potential to standardize diagnostic quality across institutions (41). In routine workflows, AI functions best as an assistive tool, preserving the pathologist’s authority while improving consistency and efficiency for complex or high-volume cases.

Beyond improving diagnostic consistency in the biopsy setting, histopathological grade is a primary driver of prognosis and treatment selection, particularly due to its strong correlation with metastatic potential (42). This link is especially critical in advanced disease. For instance, in metastatic hormone-sensitive prostate cancer (mHSPC), high Gleason scores (e.g., ISUP Grade Groups 4-5) are strongly associated with specific, aggressive metastatic patterns, including a higher burden of osseous metastases and a greater likelihood of visceral disease (43). This established biological link underscores the importance of accurate grading. Future multimodal AI models that integrate digital pathology data (Gleason patterns) with clinical data and whole-body imaging (e.g., PSMA-PET or bone scans) could therefore provide a comprehensive, automated assessment of disease burden and risk, linking primary tumor biology directly to its systemic expression (33).

### 3.3 Optimizing biopsy and early pre-treatment planning

AI also supports the step between risk identification and definitive treatment selection. “Virtual biopsy” methods use imaging-based AI to estimate histologic grade before tissue processing, offering earlier prognostic insight to guide counseling and set expectations while awaiting pathology results (38). In addition, MRI-based radiomic models provide clinically useful predictions of extraprostatic extension, with studies reporting high AUC values that exceed the subjective accuracy of conventional reads (44). These predictions contribute directly to surgical planning particularly decisions about nerve-sparing approaches and can help steer patients toward surveillance versus timely definitive intervention based on a more accurate, non-invasive assessment of grade and stage.

AI is applied at key clinical decision points in the prostate cancer pathway, starting with biopsy triage, where models integrate clinical data and imaging features to generate risk scores that help reduce unnecessary procedures while accurately identifying high-risk patients. Once a biopsy is performed, AI in digital pathology analyzes tissue slides to assign Gleason grades with expert-level accuracy, crucially reducing the inter-observer variability that can affect patient prognosis. Finally, AI assists in pre-treatment planning by using imaging-based “virtual biopsies” to predict histologic grade and radiomic models to predict cancer staging, such as extraprostatic extension, which directly informs surgical planning and the choice between surveillance or definitive intervention.

## 4 Other applications of AI in prostate cancer treatment

The impact of AI extends profoundly into the therapeutic domain, where it is being used to devise novel treatment strategies, personalize therapy selection, meticulously plan procedures like radiotherapy, and predict patient outcomes with unprecedented accuracy.

### 4.1 AI in devising adaptive treatment strategies

A major challenge in treating advanced or metastatic prostate cancer is the development of therapeutic resistance. Adaptive treatment strategies, which involve dynamically modulating therapy based on a patient’s real-time response, aim to delay or prevent this resistance. The biological rationale is to use treatment-free intervals to allow drug-sensitive cancer cells to regrow and outcompete the pre-existing resistant cells, thereby maintaining control of the tumor for a longer period. AI, and particularly deep reinforcement learning (DRL), is an ideal framework for optimizing these complex, dynamic strategies.

In a DRL model, an AI “agent” learns an optimal drug administration policy through trial and error. It interacts with a virtual patient (often a mathematical model of tumor dynamics) and learns which “actions” (e.g., give drug, withhold drug) lead to the best long-term “rewards” (e.g., maximizing time to progression, minimizing cumulative drug toxicity) (42, 45–47). Generative AI models have also been employed to design adaptive intermittent therapy schedules for androgen deprivation therapy (ADT), processing longitudinal PSA data to determine the optimal timing for treatment cycles (43). Frameworks like Intelligent Intermittent ADT are being developed to account for patient heterogeneity, deriving personalized drug schedules that prolong progression-free survival while reducing the cumulative drug dose and its associated side effects (48, 49).

### 4.2 AI for treatment recommendation and clinical decision support

For men with localized prostate cancer, the choice between treatment modalities—primarily radical prostatectomy (surgery) or radiotherapy—is complex and preference-sensitive, with different profiles of side effects. AI models are being developed as powerful clinical decision support tools to help guide these difficult conversations. By analyzing large datasets of patients with known treatments and outcomes, machine learning algorithms can predict the likelihood of success and the risk of specific side effects for an individual patient under different treatment scenarios. These models can uncover complex, non-linear relationships between patient characteristics (age, comorbidities, tumor features) and outcomes that are not captured by traditional prognostic models, thereby helping to predict which patients are likely to benefit more from surgery versus radiotherapy (50–53). Similarly, AI can forecast a patient’s likely response to systemic therapies like ADT, identifying those who may require earlier intensification of treatment (54–57).

### 4.3 AI in radiotherapy planning, delivery, and outcome prediction

Radiotherapy is a cornerstone of prostate cancer treatment that depends on the millimeter-level precision of delivering a lethal dose of radiation to the tumor while sparing surrounding healthy tissues like the bladder and rectum. This process has traditionally involved a time-consuming manual step where a radiation oncologist or dosimetrist “contours” or delineates the clinical target volume (CTV) and the organs at risk (OARs) on planning CT or MRI scans. This manual process is a major bottleneck and a significant source of inter-observer variability.

AI has revolutionized this step. AI-based auto-contouring systems, typically using CNNs, can learn from thousands of expertly contoured plans to automatically segment the prostate, CTVs, and OARs on new patient scans with extremely high speed and accuracy. This not only saves hours of physician time but also dramatically improves the consistency and standardization of treatment planning (58, 59). Recent studies show that deep learning-based auto-contouring is now achieving a level of accuracy comparable to human experts, making it suitable for clinical implementation (60). Beyond contouring, AI is central to knowledge-based treatment planning (KBP). KBP algorithms learn from a library of high-quality, previously delivered treatment plans to predict the optimal achievable dose distribution for a new patient, ensuring that the tumor is precisely targeted while minimizing dose to healthy tissues, which directly translates to fewer side effects (59). AI models are also being used to predict treatment-related toxicity before the first dose is ever delivered by analyzing planned dosimetric parameters and patient anatomy, allowing for plan modifications to mitigate risk (61–63).

### 4.4 AI-driven biomarkers for precision medicine

Perhaps one of the most exciting frontiers is the use of AI to discover novel biomarkers that can predict treatment response and prognosis from standard, routinely collected data. For example, deep learning models can analyze the morphology of cancer cells on a standard digital pathology slide and extract “AI-derived biomarkers” that are invisible to the human eye but are highly predictive of outcomes like biochemical recurrence after surgery or response to ADT. These “digital biomarkers” effectively act as a low-cost surrogate for expensive and time-consuming molecular genomic tests, providing powerful prognostic information from a simple H&E stained slide (54, 55). Multi-modal deep learning architectures are now being developed to integrate these digital pathology biomarkers with clinical, imaging, and genomic data to build comprehensive prognostic models that can predict patient outcomes with far greater accuracy than any single traditional tool (52, 53).

AI’s role in prostate cancer treatment extends to personalizing therapy and enhancing procedural precision. It is used to devise adaptive treatment strategies, such as dynamic drug schedules optimized by reinforcement learning to combat resistance and serves as a clinical decision support tool by predicting individual patient outcomes and side effects for different treatment choices like surgery versus radiotherapy. In the critical field of radiotherapy, AI revolutionizes planning through “auto-contouring,” which rapidly and consistently delineates tumors and healthy organs, and “knowledge-based planning,” which optimizes radiation dosage to minimize toxicity. Furthermore, AI is pioneering precision medicine by discovering novel “digital biomarkers” from standard pathology slides, extracting powerful prognostic information invisible to the human eye to better predict treatment response and patient outcomes.

## 5 Overarching challenges and future directions for clinical translation

Despite the immense promise and rapid progress of AI in prostate cancer management, the path from a promising algorithm in a research paper to a trusted, integrated tool in routine clinical practice is fraught with significant challenges. Overcoming these hurdles is essential for realizing the full potential of AI to improve patient care.

### 5.1 The need for rigorous clinical validation and prospective studies

A fundamental and non-negotiable requirement is rigorous and extensive clinical validation. Many published AI models report high performance on internal or retrospective datasets, but this performance often degrades significantly when the model is tested on new data from different hospitals, different scanners, or different patient populations—a problem known as poor generalizability. There is a critical need for large-scale, multi-center, prospective clinical trials to transparently evaluate and confirm the performance, safety, and clinical utility of these AI tools in real-world settings before they can be widely adopted (3, 10, 64, 65).

While AI models for prostate cancer display promising retrospective performance, there remains a pronounced gap between experimental outcomes and real-world clinical efficacy. Retrospective studies can artificially inflate accuracy due to dataset selection and methodological biases; only prospective, multi-center clinical trials can establish true clinical utility. The majority of prostate cancer AI algorithms have yet to undergo such validation, limiting their ability to influence clinical guidelines and care delivery. For implementation, multi-center prospective trials should transparently evaluate both diagnostic and prognostic capabilities in diverse populations, as demonstrated by initiatives such as the PI-CAI Challenge and digital pathology-based lymph node metastasis detection studies (21, 24, 27). Without such rigorous evidence, AI systems risk real-world failure and erosion of clinical trust. The efficacy-outcome gap must be addressed with standardized validation protocols (21, 27).

### 5.2 Addressing data equity, bias, and ensuring equitable AI deployment

The performance of any AI model is intrinsically linked to the data on which it was trained. If the training data is not diverse and representative of the full spectrum of the patient population, the resulting model can perpetuate and even amplify existing health disparities. For example, an AI model trained predominantly on data from one ethnic group may perform poorly on others due to subtle differences in anatomy, disease presentation, or imaging characteristics (66).

The risks of bias and inequity in healthcare AI are now recognized as both ethical and clinical challenges. Demographic imbalance in AI datasets can result in systematically reduced accuracy for underrepresented races, ages, genders, or socioeconomic groups, perpetuating or worsening health disparities (22, 25, 28, 31, 34, 56, 65). Studies in prostate cancer and other domains have documented disparities in AI performance across marginalized populations (31, 34, 54, 56, 62, 66).

Mitigating bias in artificial intelligence requires a coordinated approach throughout all stages of the AI lifecycle. During development, it is essential to utilize diverse and representative datasets; when certain cohorts are underrepresented or rare, synthetic data can be employed to simulate their inclusion and improve model robustness (58, 61, 64, 67). The deployment of fairness metrics—such as demographic parity, equal opportunity, and equalized odds—supports continuous monitoring and facilitates the correction of detected disparities as models are evaluated and updated (57, 59, 63, 68). Regulatory bodies have also instituted requirements for transparency, routine auditing, and post-market surveillance of AI systems, reflecting the need for ongoing accountability and continuous improvement (31, 43, 56, 66). Ultimately, the pursuit of data equity transcends technical considerations; it represents an ethical imperative to prevent the deepening of structural disparities as AI technologies become increasingly embedded in healthcare decision-making (25, 31, 54, 60).

### 5.3 The black box problem and the imperative for explainable AI

Many of the most powerful deep learning models operate as “black boxes,” with internal decision processes that are not readily interpretable by humans. This lack of transparency is a significant barrier to clinical trust, adoption, and regulatory approval. If a clinician cannot understand why an AI model made a specific recommendation particularly if it contradicts established judgment—they may hesitate to act (67). Explainable AI (XAI) is now considered a non-negotiable requirement for clinical deployment. Clinicians must be able to interpret not just outputs but also why and how a model made its decisions, especially when stakes are high for patient safety or legal liability (11, 14, 37, 44).

Recent advances in XAI have introduced a range of interpretability methods that help clarify how models reach their decisions. CAM-based approaches, such as Grad-CAM and Grad-CAM++, generate intuitive heatmaps that visually indicate which image regions were most influential in shaping the model’s output. In parallel, perturbation-based techniques like LIME and SHAP systematically alter input features to quantify their impact on the prediction. Additionally, attention-based mechanisms contribute by assigning importance weights to different features or regions within the data, providing further insights into which aspects are prioritized by the AI during inference (7, 9, 36, 38, 40, 41, 49, 52).

For prostate cancer, Grad-CAM-based visualizations have correlated with regions radiologists use to identify clinically significant cancer, and pathologists have rated these visual explanations highly for building trust in automation (7, 41, 45, 47). Large-scale reviews show that LIME achieves higher fidelity for case-level explanation, while SHAP offers consistency across features (36, 38, 40, 46). Therefore, interpretability not only builds clinician and patient confidence, but it is also increasingly required by the FDA and other regulators—now included in device approval frameworks (43–45, 50).

### 5.4 Regulatory and integration challenges

Finally, practical and regulatory hurdles abound. For an AI tool to be useful, it must be seamlessly integrated into existing clinical workflows—necessitating interoperability with PACS and EHR systems, which can be technically complex and costly. Diagnostic or therapeutic AI tools are classified as medical devices and face stringent regulatory oversight. Navigating approval pathways (such as those set by the FDA or EMA), which now require clear validation and post-market surveillance, is a time-consuming and expensive process (68). The PI-CAI challenge has provided a framework for standardized evaluation, showing that best-in-class AI systems can match radiologist performance, setting a benchmark for regulatory submissions (69).

Significant challenges hinder the clinical translation of AI in prostate cancer, starting with the critical need for rigorous validation through large-scale, prospective trials to ensure models are generalizable and effective in real-world settings, moving beyond promising retrospective results. A second major hurdle is data equity and bias, as models trained on non-diverse datasets risk perpetuating or amplifying health disparities, making the use of representative data and fairness metrics an ethical imperative. Furthermore, the “black box” nature of many algorithms erodes clinical trust, creating a non-negotiable demand for Explainable AI (XAI) to make the model’s decision-making process transparent to clinicians. Finally, practical barriers, including the technical complexity of integrating AI into existing hospital workflows (EHRs/PACS) and navigating the stringent, costly regulatory approval process for medical devices, must be overcome for widespread adoption.

## 6 Conclusion

The management of prostate cancer is undergoing a profound transformation, driven by the power of AI. AI is moving beyond the realm of research and is rapidly becoming an indispensable clinical tool. By augmenting the interpretation of medical images, AI is enabling earlier and more accurate diagnosis. By integrating complex, multimodal data, it is delivering on the promise of personalized risk stratification, helping to mitigate the long-standing problem of overtreatment. In the therapeutic setting, AI is optimizing the precision of radiotherapy, guiding complex treatment decisions, and pioneering novel adaptive strategies to combat drug resistance. The breadth of these applications, from screening to advanced therapeutics (**Table 2**), represents a significant leap forward. While formidable challenges related to robust clinical validation, data bias, interpretability, and regulatory approval must be systematically addressed, the trajectory is clear. The continued collaboration between data scientists, engineers, clinicians, and patients will undoubtedly cement AI’s role as a cornerstone of a new standard of care one that is more precise, efficient, personalized, and ultimately, more effective for every man with prostate cancer.

**Table 2:** AI applications, implications, and challenges across the prostate cancer continuum.

Clinical stage/Domain	Key AI application	Mechanistic or immunological implication	Key translational challenge
1. Screening & Triage	AI-driven risk stratification (integrating PSA, age, clinicals) (31, 32).	N/A (primary focus is statistical risk assessment).	Reducing unnecessary biopsies while preserving detection of clinically significant prostate cancer (csPCa) (23, 36). Data equity and model bias in diverse populations (66).
2. Diagnosis (Imaging)	mpMRI: Deep learning (CNNs, U-Net) for lesion detection, segmentation, and PI-RADS scoring (20–22). Ultrasound: AI-enhanced TRUS for detecting isoechoic lesions (28).	Models learn “unseen” textural patterns (radiomics) indicative of dense cellularity and altered tissue microarchitecture (31, 33).	High inter-scanner/inter-protocol variability; poor generalizability (64, 65). The “black box” problem and need for XAI to build clinician trust (67).
3. Diagnosis (Pathology)	Digital Pathology: AI-assisted Gleason grading to reduce inter-observer variability (e.g., 3 + 4 vs 4 + 3) (38–40). “Virtual Biopsy”: AI prediction of grade from imaging (38).	AI-derived “digital biomarkers” capture morphological features linked to genomic instability or cell phenotypes, acting as a surrogate for molecular tests (54, 55).	Workflow integration with Laboratory Information Systems (LIS) and EHRs. Standardization of whole-slide imaging. Regulatory approval as a diagnostic device (68).
4. Treatment (Local)	Radiotherapy: AI-based auto-contouring of organs-at-risk (OARs) and clinical target volumes (CTVs) (58, 59). Knowledge-Based Planning (KBP) for dose optimization (59).	N/A (primary focus is physics and geometry optimization to spare healthy tissue).	Ensuring robustness of auto-contouring across anatomical variations (60). Clinical acceptance and integration into treatment planning systems (58).
5. Treatment (Advanced)	Adaptive Therapy: Deep reinforcement learning (DRL) to optimize intermittent Androgen Deprivation Therapy (ADT) schedules (46, 48). Prognostication: Multimodal models (imaging + path + genomics) to predict treatment response/toxicity (52, 53).	DRL models learn tumor evolutionary dynamics (e.g., competition between drug-sensitive and resistant cell populations) (46). Digital biomarkers act as surrogates for molecular pathways (e.g., AR signaling, DNA repair) (54, 55).	Need for robust “virtual patient” models to train DRL agents (45, 47). Data heterogeneity in multimodal models. Ethical and regulatory hurdles for AI-driven treatment decisions (68).
Clinical Stage/Domain	Key AI Application	Mechanistic or Immunological Implication	Key Translational Challenge
1. Screening & Triage	AI-driven risk stratification (integrating PSA, age, clinicals) (31, 32).	N/A (primary focus is statistical risk assessment).	Reducing unnecessary biopsies while preserving detection of clinically significant prostate cancer (csPCa) (23, 36). Data equity and model bias in diverse populations (66).
2. Diagnosis (Imaging)	mpMRI: Deep learning (CNNs, U-Net) for lesion detection, segmentation, and PI-RADS scoring (20–22). Ultrasound: AI-enhanced TRUS for detecting isoechoic lesions (28).	Models learn “unseen” textural patterns (radiomics) indicative of dense cellularity and altered tissue microarchitecture (31, 33).	High inter-scanner/inter-protocol variability; poor generalizability (64, 65). The “black box” problem and need for XAI to build clinician trust (67).
3. Diagnosis (Pathology)	Digital Pathology: AI-assisted Gleason grading to reduce inter-observer variability (e.g., 3 + 4 vs 4 + 3) (38–40). “Virtual Biopsy”: AI prediction of grade from imaging (38).	AI-derived “digital biomarkers” capture morphological features linked to genomic instability or cell phenotypes, acting as a surrogate for molecular tests (54, 55).	Workflow integration with Laboratory Information Systems (LIS) and EHRs. Standardization of whole-slide imaging. Regulatory approval as a diagnostic device (68).
4. Treatment (Local)	Radiotherapy: AI-based auto-contouring of organs-at-risk (OARs) and clinical target volumes (CTVs) (58, 59). Knowledge-Based Planning (KBP) for dose optimization (59).	N/A (primary focus is physics and geometry optimization to spare healthy tissue).	Ensuring robustness of auto-contouring across anatomical variations (60). Clinical acceptance and integration into treatment planning systems (58).
5. Treatment (Advanced)	Adaptive Therapy: Deep reinforcement learning (DRL) to optimize intermittent Androgen Deprivation Therapy (ADT) schedules (46, 48). Prognostication: Multimodal models (imaging + path + genomics) to predict treatment response/toxicity (52, 53).	DRL models learn tumor evolutionary dynamics (e.g., competition between drug-sensitive and resistant cell populations) (46). Digital biomarkers act as surrogates for molecular pathways (e.g., AR signaling, DNA repair) (54, 55).	Need for robust “virtual patient” models to train DRL agents (45, 47). Data heterogeneity in multimodal models. Ethical and regulatory hurdles for AI-driven treatment decisions (68).
